# Supplementary material for: Harmful cultural practices during perinatal period and associated factors among women of childbearing age in Southern Ethiopia: Community based cross-sectional study
Source: PLoS One. 2021 Jul 2;16(7):e0254095. doi: 10.1371/journal.pone.0254095 (PMC8253409; doi:10.1371/journal.pone.0254095)
Supplement: S1 File — (PDF) [file pone.0254095.s001.pdf]

English Version Questionnaire

Interview Record for Quantitative Data   Identification Number [ ]

Cultural malpractice during perinatal period and associated factors among women of childbearing age in Southern Ethiopia: 2019/20

S.No.	Question	Response	Skip
**Part I: Socio- demographic Characteristics**			
101	Age of the mother	/____________/ in complete year	
102	Religion	1. Orthodox<br>2. Catholic<br>3. Protestant<br>4. Other, Specify___________	
103	Ethnicity	1. Gurage<br>2. Amhara<br>3. Oromo<br>4. Tigray<br>5. Other, Specify____________	
104	Occupational status:	1.House wife<br>2.Gove'temployee<br>3.Merchant<br>4. Student<br>5.Other (specify)	
105	Marital status	1. Married<br>2. Divorce<br>3. Widowed	
106	Place of residence	1. Urban<br>2. Rural	

107	Educational status of mother	1. Cannot able to read and write<br>2. Can read and write<br>3. Grade 1-8<br>4. Grade 9-12<br>5. College and above	
108	Average monthly income	/________________/ in ETH birr	
**Part II: Obstetrics characteristics of study participants**			
201	How many delivery have you ever had	------------------------- in number	
202	Who attend your last childbirth?	1. Family<br>2. Neighbors<br>3. Relatives<br>4.Trained traditional birth attendant<br>5. Other	
203	Do you have ANC follow up during the last childbirth?	1. Yes<br>2. No. if you say “yes”	
204	Where you have taken antenatal care?	1.Goverment Health facility<br>2.Private Health facility	
205	Travel time to health institution	______ [KM]?	
**III**	**Cultural mal practice during perinatal period**		
301	Did you make any food prohibition during your pregnancy?	1. Yes<br>2. No	
302	If yes, specify the type of food prohibited	________________________	
303	Did you use abdominal massage with butter during labour?	1. Yes<br>2. No	

304	Have you drink kosso during pregnancy?	1. Yes<br>2. No	
305	Have you drink telba during pregnancy?	1. Yes<br>2. No	
306	Where did you deliver your last child?	1. Home<br>2. Health facility	
307	What instrument used to cut cord?	1. Boiled new blade<br>2.Unclean blade used before<br>**3.** UN boiled new blade<br>4.Boiled blade used before	
308	Was the umbilical cord tied?	1. Yes<br>2. No	
308	If yes, what type of thread do you use?	1. Boiled thread<br>2.unclean thread<br>3.other	
309	What was given for the new born body immediately after birth?	1. Butter<br>2.Cow milk<br>3. Water<br>4.nothing	
310	When the child was started breast milk?	1. Immediately (within1 hour)<br>2. Within 1-24hours<br>3.After 24 hours<br>4.other (specify)____________	
311	Did you give colostrum to new born?	1. Yes<br>2. No	
312	When did you wash the new born after delivery	1. Immediately (with in 1 hour)<br>2. Within 1-24 hour<br>3. After 24 hour<br>4. Other Specify ___________	
313	Did you have any health	1. Yes	

	problem?	2. No	
314	If yes, where did you get sick help?	1. Health institution<br>2. Religious<br>3. Traditional healer<br>4. Other (specify)____	
315	How long after birth did you first put the baby on the breast?	1. Immediately after delivery<br>2. Within one hour after delivery<br>3. After one hour of delivery	
316	Did you give the first liquid (colostrum) that came out from your breasts?	1. Yes<br>2. No	
317	What did you do with the first liquid (colostrum)?	________________________	
317	Did you give anything to drink other than breast milk in the first three days after birth?	1. Yes<br>2. No	
318	If the above answer is "Yes", which fluid you gave?	1. Plain water<br>2. Butter<br>3. Animal Milk<br>4. Honey<br>5. Other, Specify____________	

Amharic Version Questionnaire

ክፍል አንድ: የማህበራዊና ስነህዝባዊ ስብጥር መረጃ

1. እድሜ?______________________ በአመት

2 እምነት፦ 1□ሙስሊም 2□ ኦርቶዶክስ 3□ ፕሮቴስታንት 4□ ሌላ

3. ብሄር፦ 1□ኦሮሞ 2□አማራ 3. ትግሬ 4□ጉራጌ 5□ሌላ

4. የስራ ሁኔታ፦1□የቤት እመቤት 2□ የመንግስት ሰራተኛ 3□ ነጋዴ 4□ተማሪ 5□ሌላ
5. የትዳር ሁኔታ፦ 1□ ያገባች 2□ያላገባች 3□የተፋታች 4□ባሏ የሞተባት 5 □ሌላ

6□የትምህርት ሁኔታ፦ 1□ ማንበብ እና መፃፍየምችል 2. የመጀመሪያ ደረጃ 3□ሁለተኛ ደረጃ 4 □መሰናዶ እና ከዛ በላይ

7. የቤተሰብ ወራዊ ገቢ ______ብር
8. ጤና ተቋም ለመህደ ስንት ምን ያክል ይፈጅብሻል ________Minute
ክፍል ሁለት: የስነ ተዋልዶ ክፍል

9. ስንት ጊዜ አርግዘሻል 1.____________

10. ስንት ጊዜ ወልደሻል 1.____________

11. ክትትል አለሽ 1□ አዎ 2□የለም

ክፍል ሶስት: የባህላዊ ልማድ እርግዝና ጊዜ

12. በእርግዝና ጊዜ የባህላዊ ልማድ ታደረጊለሽ 1□ አዎ 2□የለም

13. በእርግዝና ወቅት ምግብ ይከለክልሽ ነበር? 1□ አዎ 2□የለም

14. አዎ ከሆነ የከለከለሽን የምግብ አይነት ዝርዝር

15. በእርግዝና ወቅት ያጋጠመሽ የጤና ችግር ነበረብሽ? 1□አዎ 2□የለም

16. አዎ ከሆነ ከየት ነው አገልግሎት የምታገኚው? 1. ከጤና ተቋአም 2. ልምድ በሌላት ባህላዊ አዋላጅ 3. ልምድ ባላት ባህላዊ አዋላጅ 4.ከባቅህላዊ መድሃኒተኛ 5. ሌላ

17. ምን አይነት የባህላዊ ልምድ ታደረጊለሽ? 1. ሆድ ማሸት 2□ተልባ መጠጣት 3□ኮሶ መጠጣት 4□ሌላ

ክፍል አራት፡ በወሊድ ጊዜ የሚደረጉ ባህላዊ ልማዶች
18. በወሊድ ጊዜ የባህላዊ ልምድ ታደረጊለሽ? 1.አዎ 2□የለም
19. አዎ ካልሽ ምን አይነት? 1. ማህጸን ማሸት 2□ ሆድ ማሸት 3. ሌላ
20. ከላይ የተጠቀሱትን ባህላዊ ልማዶች ልምድ አለሽ? 1□ አዎ 2□ የለም
21. አዎ ካልሽ ምን አይነት? 1□ሆድ ማሸት 2□ማህፀን ማሸት 3□ሌላ
22. የመጨረሻ ልጅሽን የተገላገልሽው የት ነው? 1□ ቤት 2□ የጤና ተቋም
23. ቤት ካልሽ ማን ተከታተለሽ? 1□ቤተሰብ 2□ጎረቤት 3□ሌላ

24. እትብት ለመቁረጥ ምን ተጠቀምሽ? 1□ የተፈላ አዲስ ምላጭ 2□ከዚህ በፊት ያገለገለ ያልፀዳ ምላጭ 3□ የተፈላ አዲስ ምላጭ 4□ ከዚህ በፊት ያገለገለ የተፈላ ምላጭ 5□ሌላ
25. የእንግዴ ልጅ ለማስወጣት ምን አይነት ዘዴ ተጠቀምሽ 1□ሳይታሰብ በድንገት 2□በማወዝወዝ/በማንቀጥቀጥ 3□ማህፀን በማሸት 4□ በጉሮሮ ድምጽ በማሰማት 5. እንግዴ ልጁን በማሰር ከሆድ መለየት 6□ሌላ
26. እትብቱ ተያይዞ ነበር? 1□ አዎ 2□ አይ

27. አዎ ካልሽ ምን አይነት ክር ተጠቀምሽ? 1. የተፈላ ክር 2□ ያልተጸዳ ክር   3□ሌላ
28. ምን አይነት ልምድ ነበር የተጠቀምሽው እትብቱን ለመቁረጥ?

1.   ምንም   2□ የላም ፍግ   3□   ቅቤ   4.ሌላ

ክፍል አምስት፡ ባህላዊ ልማድ ከወሊድ በኂላ

በወሊድ በኂላ የ ባህላዊ ልምድ ታደረጊለሽ? 1.አዎ 2□የለም

29. አዲስ ለተወለደው ልጅ ልክ እንደተወለደ ወዲያውኑ ምን ይሰጠዋል?

1. ቅቤ   2□ የላም ወተት   3□   ውሃ   4□   ምንም   5□ሌላ

30. መቼ ነው ልጁ ጡት መጥባት የሚጀምረው?

1.ወዲያውኑ (በ 1 ሰአት ውስጥ)   2□ከ 1-24 ሰአት ውስጥ   3□   ከ 24 ሰአት በኂላ
4□ሌላ

31. አዲስ ለተወለደው ልጅ እንገር ትሰጪዋለሽ?   1 □አዎ   2□   የለም

32. አይ ካልሽ ለምን?

33. ልጁ ከተወለደ በኂላ መቼ ይታጠባል?   1□ ወዲያውኑ(በ 1 ሰአት ውስጥ)   2□
ከ 1-24 ሰአት በኂላ   3□ከ 1-24 ሰአት ውስጥ   4□ሌላ

34. የጤና ችግር ነበረብሽ?   1. አዎ   2□   የለም

35. አዎ ካልሽ እርዳታ ከየት አገኘሽ? 1. ከጤና ተቋም   2□ከእምነት   3□   ከባህላዊ
መድሃኒተኛ 4□ሌላ

ለነበረን ቆይታ አመሰግናልሁ !!!
