# Supplementary material for: Harmful cultural practices during perinatal period and associated factors among women of childbearing age in Southern Ethiopia: Community based cross-sectional study
Source: PLoS One. 2021 Jul 2;16(7):e0254095. doi: 10.1371/journal.pone.0254095 (PMC8253409; doi:10.1371/journal.pone.0254095)
Supplement: Attachment — Submitted filename: PONE-D-20-34446_reviewer.08.12.2020.pdf [file pone.0254095.s003.pdf]

PLOS ONE

Cultural malpractice during perinatal period and associated factors among women of child bearing age in Southern Ethiopia: Community based cross-sectional study

--Manuscript Draft--

**Manuscript Number:**	PONE-D-20-34446
**Article Type:**	Research Article
**Full Title:**	Cultural malpractice during perinatal period and associated factors among women of child bearing age in Southern Ethiopia: Community based cross-sectional study
**Short Title:**	Cultural malpractice during perinatal period and associated factors
**Corresponding Author:**	Haimanot Abebe<br>Wolkite University<br>Wolkite, ETHIOPIA
**Keywords:**	Cultural malpractice; harmful traditional practice; pregnancy; childbirth; postnatal; perinatal
**Abstract:**	Introduction<br><br>Even though maternal mortality ratio dropped by 38% in the last decade, 810 women died from preventable causes related to pregnancy and childbirth every day, and two-thirds of maternal deaths occur in Sub-Saharan Africa alone. Skilled care before, during and after childbirth can save the lives of women and newborns. The main factor that prevent women from receiving care during pregnancy and childbirth is cultural beliefs and practices. The objective of this study is to assess the level of cultural malpractices during pregnancy, childbirth and postnatal period, and associated factors among women of childbearing age in Southern Ethiopia.<br><br>Methods<br><br>Community based cross sectional study design was conducted in Gurage zone, among calculated representative sample of 422 women of reproductive age group who have at least one history of childbirth. Data was collected by six experienced and trained data collectors using pretested structured questionnaire with face to face interview. Perinatal period is considered as time period including the pregnancy, childbirth and postnatal period. Cultural malpractice is assessed using 11 questions and those who participated in any one of them is considered as cultural malpractice. The data were entered into Epi Info version ™ 7 and exported to Statistical Package for Social Science (SPSS) version 25 for cleaning and analysis.<br><br>Results<br><br>Overall cultural malpractice was found to be 71.4% [95%CI, 66.6-76.0]. Had no formal education 3.79 [AOR 3.79, 95%CI, 1.97-7.28], being rural residence 4.41 [AOR 4.41, 95%CI, 2.63-7.39], had no antenatal care in the last pregnancy 2.62 [AOR 2.62, 95%CI, 1.54-4.48], attended by untrained attendants 2.67 [AOR 2.67, 95%CI, 1.58-4.51] were significantly associated with cultural malpractices during perinatal period.<br><br>Conclusion<br><br>Cultural malpractice was found to be high in the study area. In this study, had no formal education, being a rural residence, had no ANC follow up for the last pregnancy and attended by untrained attendant were an independent factors for cultural malpractice during perinatal period. Thus, strong multi-sectoral collaboration targeted at improving women educational status and primary health care workers should take up the active role of women health education on the importance of ANC visit are recommended.
**Order of Authors:**	Haimanot Abebe
	Girma Alemayehu
	Berhanu Semere

Powered by Editorial Manager® and ProduXion Manager® from Aries Systems Corporation

Additional Information:	
**Question**	**Response**
**Financial Disclosure**<br><br>Enter a financial disclosure statement that describes the sources of funding for the work included in this submission. Review the submission guidelines for detailed requirements. View published research articles from *PLOS ONE* for specific examples.<br><br>This statement is required for submission and **will appear in the published article** if the submission is accepted. Please make sure it is accurate.<br><br>**Unfunded studies**<br>Enter: *The author(s) received no specific funding for this work.*<br><br>**Funded studies**<br>Enter a statement with the following details:<br>• Initials of the authors who received each award<br>• Grant numbers awarded to each author<br>• The full name of each funder<br>• URL of each funder website<br>• Did the sponsors or funders play any role in the study design, data collection and analysis, decision to publish, or preparation of the manuscript?<br>• **NO** - Include this sentence at the end of your statement: *The funders had no role in study design, data collection and analysis, decision to publish, or preparation of the manuscript.*<br>• **YES** - Specify the role(s) played.<br><br>* typeset	The funders had no role in study design, data collection and analysis, decision to publish, or preparation of the manuscript.
**Competing Interests**<br><br>Use the instructions below to enter a competing interest statement for this submission. On behalf of all authors, disclose any competing interests that could be perceived to bias this work—acknowledging all financial support and any other relevant financial or non-financial competing interests.	he authors have declared that no competing interests exist.

Powered by Editorial Manager® and ProduXion Manager® from Aries Systems Corporation

This statement **will appear in the published article** if the submission is accepted. Please make sure it is accurate. View published research articles from *PLOS ONE* for specific examples.<br><br>**NO authors have competing interests**<br><br>Enter: *The authors have declared that no competing interests exist.*<br><br>**Authors with competing interests**<br><br>Enter competing interest details beginning with this statement:<br><br>*I have read the journal's policy and the authors of this manuscript have the following competing interests: [insert competing interests here]*<br><br>* typeset	
**Ethics Statement**<br><br>Enter an ethics statement for this submission. This statement is required if the study involved:<br><br>• Human participants<br>• Human specimens or tissue<br>• Vertebrate animals or cephalopods<br>• Vertebrate embryos or tissues<br>• Field research<br><br>Write "N/A" if the submission does not require an ethics statement.<br><br>General guidance is provided below. Consult the submission guidelines for detailed instructions. **Make sure that all information entered here is included in the Methods section of the manuscript.**	Ethical clearance was obtained from the Wolkite University College of Health and Medical Science Institutional Health Research Ethical Review Committee. An official letter was sent to the Gurage health office and the data collection was begun after permission and cooperation letter was written to all districts on which the study was carried out. The study, purpose, procedure and duration, rights of the respondents and data safety issues, possible risks and benefits of the study were clearly explained to each participant using the local language. Then, all subjects gave their informed written consent for inclusion before they participated in the study.

**Format for specific study types**<br><br>**Human Subject Research (involving human participants and/or tissue)**<br>• Give the name of the institutional review board or ethics committee that approved the study<br>• Include the approval number and/or a statement indicating approval of this research<br>• Indicate the form of consent obtained (written/oral) or the reason that consent was not obtained (e.g. the data were analyzed anonymously)<br><br>**Animal Research (involving vertebrate animals, embryos or tissues)**<br>• Provide the name of the Institutional Animal Care and Use Committee (IACUC) or other relevant ethics board that reviewed the study protocol, and indicate whether they approved this research or granted a formal waiver of ethical approval<br>• Include an approval number if one was obtained<br>• If the study involved *non-human primates*, add *additional details* about animal welfare and steps taken to ameliorate suffering<br>• If anesthesia, euthanasia, or any kind of animal sacrifice is part of the study, include briefly which substances and/or methods were applied<br><br>**Field Research**<br><br>Include the following details if this study involves the collection of plant, animal, or other materials from a natural setting:<br>• Field permit number<br>• Name of the institution or relevant body that granted permission	
**Data Availability**<br><br>Authors are required to make all data underlying the findings described fully available, without restriction, and from the time of publication. PLOS allows rare exceptions to address legal and ethical concerns. See the PLOS Data Policy and FAQ for detailed information.	No - some restrictions will apply

Powered by Editorial Manager® and ProduXion Manager® from Aries Systems Corporation

A Data Availability Statement describing where the data can be found is required at submission. Your answers to this question constitute the Data Availability Statement and **will be published in the article**, if accepted.<br><br>**Important:** Stating 'data available on request from the author' is not sufficient. If your data are only available upon request, select 'No' for the first question and explain your exceptional situation in the text box.<br><br>Do the authors confirm that all data underlying the findings described in their manuscript are fully available without restriction?	
**Describe where the data may be found in full sentences. If you are copying our sample text, replace any instances of XXX with the appropriate details.**<br><br>• If the data are **held or will be held in a public repository**, include URLs, accession numbers or DOIs. If this information will only be available after acceptance, indicate this by ticking the box below. For example: *All XXX files are available from the XXX database (accession number(s) XXX, XXX.).*<br>• If the data are all contained **within the manuscript and/or Supporting Information files**, enter the following: *All relevant data are within the manuscript and its Supporting Information files.*<br>• If neither of these applies but you are able to provide **details of access elsewhere**, with or without limitations, please do so. For example:<br><br>*Data cannot be shared publicly because of [XXX]. Data are available from the XXX Institutional Data Access / Ethics Committee (contact via XXX) for researchers who meet the criteria for access to confidential data.*<br><br>*The data underlying the results presented in the study are available from (include the name of the third party*	All relevant data are within the manuscript and its Supporting Information files.

and contact information or URL).<br>• This text is appropriate if the data are owned by a third party and authors do not have permission to share the data.<br><br>* typeset	
Additional data availability information:	

Click here to access/download;Manuscript;CMP_ main manuscript Plose one.docx

Cultural malpractice during perinatal period and associated factors among women of child bearing age in Southern Ethiopia: Community based cross-sectional study

Haimanot Abebe[1], Girma Alemayehu[1], Berhanu Semere[1]

[1]. Department of Public Health, College of Health Sciences and Medicine, Wolkite University, Wolkite, Ethiopia.

[*]Corresponding author

E-mail: haimanotabebe78@gmail.com

Abstract

Introduction: Even though maternal mortality ratio dropped by 38% in the last decade, 810 women died from preventable causes related to pregnancy and childbirth every day, and two-thirds of maternal deaths occur in Sub-Saharan Africa alone. Skilled care before, during and after childbirth can save the lives of women and newborns. The main factor that prevent women from receiving care during pregnancy and childbirth is cultural beliefs and practices. The objective of this study is to assess the level of cultural malpractices during pregnancy, childbirth and postnatal period, and associated factors among women of childbearing age in Southern Ethiopia.

Methods: Community based cross sectional study design was conducted in Gurage zone, among calculated representative sample of 422 women of reproductive age group who have at least one history of childbirth. Data was collected by six experienced and trained data collectors using pretested structured questionnaire with face to face interview. Perinatal period is considered as time period including the pregnancy, childbirth and postnatal period. Cultural malpractice is assessed using 11 questions and those who participated in any one of them is considered as cultural malpractice. The data were entered into Epi Info version ™ 7 and exported to Statistical Package for Social Science (SPSS) version 25 for cleaning and analysis.

Results: Overall cultural malpractice was found to be 71.4% [95%CI, 66.6-76.0]. Had no formal education 3.79 [AOR 3.79, 95%CI, 1.97-7.28], being rural residence 4.41 [AOR 4.41, 95%CI, 2.63-7.39], had no antenatal care in the last pregnancy 2.62 [AOR 2.62, 95%CI, 1.54-4.48], attended by untrained attendants 2.67 [AOR 2.67, 95%CI, 1.58-4.51] were significantly associated with cultural malpractices during perinatal period.

Conclusion: Cultural malpractice was found to be high in the study area. In this study, had no formal education, being a rural residence, had no ANC follow up for the last pregnancy and attended by untrained attendant were an independent factors for cultural malpractice during perinatal period. Thus, strong multi-sectoral collaboration targeted at improving women educational status and primary health care workers should take up the active role of women health education on the importance of ANC visit are recommended.

Key words*:* Cultural malpractice, harmful traditional practice, pregnancy, childbirth, postnatal, perinatal.

Introduction

Even though maternal mortality ratio dropped by 38% in the last decade, daily 810 women died from preventable causes related to pregnancy and childbirth, 94% of them occur in low and middle-income countries and two-thirds of maternal deaths occur in Sub-Saharan Africa alone. Ethiopia is one of the countries with the highest maternal mortality; according to the 2016 Ethiopian Demographic and Health survey report pregnancy related mortality ratio was 412 maternal deaths per 100,000 live births. Skilled care before, during and after childbirth can save the lives of women and newborns. The main factor that prevent women from receiving care during pregnancy and childbirth is cultural beliefs and practices among others [1, 2].

Cultural malpractice during perinatal period which includes pregnancy, childbirth and postnatal period refer to deep-rooted traditional practices which adversely affect physical, sexual, psychological well-being, and/ or violate human rights, socio-economic participation and benefits of women, children and societies at large. The types and prevalence of these practices vary among regions, cultural settings, religious values and cultural heritages [3, 4].

In developing countries cultural belief and practice may avert women from accessing antenatal, delivery and postnatal care. It also has significant influence on the place of delivery and increases the probability of home delivery [5-10]. In most part of Africa food choice and preference of pregnant women is shaped by the cultural beliefs and taboos. As a result, there are varieties of nutritious food items that are avoided during pregnancy and postnatal period increasing vulnerability of the women for malnutrition [11, 12].

According to the 2016 Ethiopian Demographic and Health survey, only 32% of women had four or more antenatal care visits during their pregnancy, 73% of pregnant women gave birth at home and 81% did not receive postnatal checkup. Access to antenatal care, delivery and postnatal care increase with women’s education and empowerment [2].

Cultural malpractice is common among women and children in Ethiopia. Surveys conducted in Ethiopia indicated, the prevalence of cultural malpractice during pregnancy ranges from 37-85%. The commonly mentioned cultural malpractice includes the food restriction and taboos, abdominal and uterine massage, home delivery, avoiding colostrum, cutting umbilical cord by unsterile sharp materials, delaying initiation of breast feeding, early bath, giving butter and/or

water for new born, using of koso (traditional herb). Maternal age, women's empowerment, educational status of the women, parity of the women, societal awareness, religion, low economic status of women and girls, imbalanced gender relations and distance from health facility were among the factors identified to affect the cultural malpractice [2, 4, 12-18].

In order to improve maternal health, barriers that limit access to quality maternal health services must be identified and addressed, one of the barriers being cultural malpractice [1]. In Ethiopia, data on cultural practice during pregnancy, child birth and postnatal period is not well understood and no study was conducted in the study setting to assess the problem. Thus, this study attempts to determine the prevalence of cultural malpractice during pregnancy, childbirth & postnatal period and associated factors among reproductive age women in Southern Ethiopia.

The findings of the study could have a potential relevance and significance to understand the magnitude of the problem, make evidence- based decisions and take appropriate actions by developing health care plan, policy and programs to resolve the problem.

Methods

Study area and period

The study was conducted in the selected woredas of Gurage zone, Southern Ethiopia. According to the data obtained from the zonal administration, there are thirteen woredas and three town administration in the zone. According to 2017 Ethiopian central statistical agency population projection, the total population of Gurage zone is 1,635,311; of 842,065 are females and the remaining 793,246 are males [19]. There are seven hospitals (five public and two non-governmental) serving the total population in the zone. Five of the hospitals in the zone are primary hospitals and the remaining two is general zonal hospital. All hospitals provide comprehensive emergency obstetric care services. Additionally there are 72 health centers which provide basic emergency obstetric care services in Gurage zone. The study was conducted from April to May, 2019.

Study design and population

Community based cross sectional study design was conducted among women of reproductive age group (15-49 years.) who have at least one history of childbirth and lived in the area for the last six months.

Sample size determination

The sample size for the study was calculated using Epi Info™ version 7 StatCalc function of Sample Size calculation for population survey at 95% confidence interval (CI), 5% marginal of error, considering 50.9% of mothers had cultural malpractices during their pregnancy from related study in Meshenti town, west Gojam zone, North West Ethiopia [18] which gave the largest sample size and adding 10% non-response rate, a total of 422 study participants were estimated for this study.

Sampling technique

From the woredas of the zone, five of them and one town administration were selected by simple random sampling technique using lottery method. Then, three kebeles from each woredas and two kebeles from Butajira town were randomly selected. Households with pregnant women were listed out from family folder of health extension workers (HEW) and the study participants were selected using simple random sampling technique with Excel generated random numbers. Total sample size was allocated proportionally to the selected kebeles and town based on the number of pregnant women in their respective kebeles.

Data collection techniques and procedure

Data were collected by six experienced and trained data collectors who are Bachelor degree holders using structured questionnaire with face to face interview. In addition, two experienced supervisors were used to guide the data collection process. After reviewing relevant literatures from previous related studies and other materials, the questionnaire was prepared in English, translated to local language (Amharic) and administered with the Amharic version to facilitate understanding. One day training was provided for the data collectors and the supervisors, the questionnaire was pretested a week before the actual survey in comparable setting in Silte town on 5% of the calculated sample size, after which the necessary correction and modification was

made accordingly. The filled questionnaires were checked on daily basis for their completeness and internal consistency.

Operational definitions

Perinatal period: Time period including the pregnancy, childbirth and postnatal period.

Cultural malpractice: having practiced any one of the following is considered as cultural malpractice.

- Food taboos, Avoiding colostrum, Delay initiation of breast feed, giving butter and/or water for new born(prelacteal feeding), Use of kosso and Use of telba
- Abdominal massage
- Cutting umbilical cord by unsterile sharp materials, tie umbilical cord by unclean materials
- Home delivery, Early bath

Data processing and analysis

The data collected were entered into Epi Info version ™ 7 and exported to Statistical Package for Social Science (SPSS) version 25 for cleaning and analysis. Descriptive statistics were performed and the findings were presented with text and tables. Binary logistic regression was used to assess the association between each independent variables and outcome variable. Hosmer-Lemeshow statistic and Omnibus tests was done for model fitness. All variables with $P<0.25$ in the bivariate analysis were included in the final model of multivariate analysis in order to control all possible confounders. In addition, variables which were significant in previous studies and from context point of view included in the final model even if the above criteria was not meet. A variance inflation factor >10 and standard error >2 were considered as suggesting the existence of multi co-linearity. The direction and strength of statistical association was measured by an odds ratio with 95% CI. Adjusted odds ratio along with 95%CI was estimated to identify factors for cultural malpractice. In this study P-value < 0.05 was considered to declare a result as a statistically significant association.

Ethical consideration

Ethical clearance was obtained from the Wolkite University College of Health and Medical Science Institutional Health Research Ethical Review Committee. An official letter was sent to

the Gurage health office and the data collection was begun after permission and cooperation letter was written to all districts on which the study was carried out. The study, purpose, procedure and duration, rights of the respondents and data safety issues, possible risks and benefits of the study were clearly explained to each participant using the local language. Then, all subjects gave their informed written consent for inclusion before they participated in the study.

Result

Socio- demographic characteristics of study participants

A total of 413 study participants were involved in this study, making response rate of 97.9%. The mean age of study participants were 27.6 (SD ± 5.4 years). More than half, 232(56.2%) of respondents were Orthodox followers by religion. More than four-fifth, 342 (82.8%) of the participant were Gurage by ethnicity. Almost two-third, 278(67.3%) of the participant were from the rural areas. Almost three-fifth, 278 (60.5%) of the respondents had no formal education. Nearly three-fourth, 299 (72.4%) of the study participants were self-employed by their occupation. Nearly two-third, 268(64.9%) of the respondents had ≥ 1000 Ethiopian birr average monthly income (See Table 1).

Table 1: Socio- demographic characteristics of study participants in Southern, Ethiopia, 2019 (n=413).

Variable	Frequency	Percent
**Age (Year)**		
15-24	115	27.8
25-34	242	58.6
≥ 35	56	13.6
**Ethnicity**		
Gurage	342	82.8
Amhara	22	11.9
Oromo	49	5.3
**Marital status**		

Married	385	93.2
Divorced	17	4.1
Windowed	11	2.7
**Residence**		
Rural	278	67.3
Urban	135	32.7
**Education**		
No formal education	250	60.5
Primary Education	89	21.6
Secondary and above	74	17.9
**Occupation**		
Self-employed	299	72.4
Government Employed	114	27.6
**Average monthly income**		
≤500	41	9.9
501-999	104	25.2
≥1000	268	64.9

Obstetrics characteristics of study participants

Nearly three-fifth, 227(55.0%) of the respondents were given 2-4 childbirth. Almost half, 216(53.7%) of the participants were attended by trained health professional during the last child birth. Nearly three-fifth, 229(55.4%) of the participant had ANC follow up during the last pregnancy. More than three-fourth, 187(81.7%) of the participants had taken antenatal care in government health facility. Almost half, 213(51.6%) of the participants had taken less than 5km to reach in nearby health facility.

Cultural malpractice during perinatal period

In this study, 295 (71.4%) of participants were performed cultural malpractice during perinatal period. Regarding food taboos, 183(44.3%) of the participants had consumed food taboos.

Nearly one-fourth, 102(24.7%) of the participants applied abdominal massage with butter to facilitate labor. Nearly half, 193(46.7 %) of the participants were drunk koso during pregnancy. Nearly half, 198(47.9%) of the participants were drunk telba during pregnancy. Concerning birth place, 197 (47.7%) of participants were assisted by untrained TBA at home. Regarding umbilical cord care, 184 (44.6%) of respondents used unclean blade to cut umbilical cord and 174 (42.1%) of them used unclean thread to tie umbilical cord. Regarding breast feeding, 146(35.4%) of the participants were gave pre lacteal feeding (butter, honey, sugar and water). Nearly one-third, 131(31.7%) of the participants discarded the colostrum (first yellowish milk). Nearly one-third, 120(29.1%) of the participants were not gave breast feeding within the first hour of birth. Regarding initial time of bathing, 161(39.0%) of the participants were not washed their babies within 24hr of birth (See Table 2).

Table 2: Cultural malpractice during perinatal period in Southern Ethiopia, 2019 (n = 413).

Variable	Frequency	Percent
**Food taboos**		
No	240	55.7
Yes	183	44.5
**Abdominal massage with butter**		
No	311	75.3
Yes	102	24.7
**Koso drinking**		
No	220	53.3
Yes	193	46.7
**Telba drinking**		
No	215	52.1
Yes	198	47.9
**Place of Birth**		
Home	197	47.7
Health facility	216	52.3
**Instrument used to cut the umbilical cord**		
Unclean blade	184	55.4
Clean blade	229	44.6
**Material used to tie cord**		
Unclean	174	42.1
Clean	239	57.9
**Pre lacteal feeding**		
No	146	
Yes	267	
**Colostrum feeding**		
No	131	31.7

Yes	282	68.3
**Time to start breastfeeding**		
Not within 1hr	120	29.1
Within 1hr	293	70.9
**Initial time of bathing**		
Not within 24hr	161	39.0
Within 24hr	252	61.0

Factors associated with cultural malpractice during perinatal period

Multivariable analysis showed that the odd of cultural malpractice during perinatal period were almost four [AOR 3.79, 95%CI, 1.97-7.28] times higher in woman who had no formal education than those who had secondary education and above. Participants who were rural by residence were nearly five [AOR 4.41, 95%CI, 2.63-7.39] times more likely performed cultural malpractice during perinatal period than those who were urban by residence. Regarding ANC follow up, participants who had ANC follow up during the last pregnancy were almost three [AOR 2.62, 95%CI, 1.54-4.48] times more likely execute cultural malpractice during perinatal period. Participants who were attended by untrained attendant during the last child birth were almost three [AOR 2.67, 95%CI, 1.58-4.51] times more likely performed cultural malpractice than those who were attended by trained attendant **(See Table 3).**

Table 3: Factors associated with cultural malpractice during perinatal period in Sothern Ethiopia, 2019 (N=413).

**Variables**	**Cultural Malpractice**		**COR (95%)**	**AOR (95%)**
	**Yes (%)**	**No (%)**		
**Educational status**				
No formal education	200(67.8)	50(42.4)	3.05(1.75-5.31)	**3.79(1.97-7.28)***
Primary education	53(18.0)	36(30.5)	1.12(0.60-2.09)	0.93(0.45-1.95)
Secondary education and above	42(14.2)	32(27.1)	1.00	1.00
**Residences**				
Rural	226(76.6)	52(44.1)	4.16(2.64-6.54)	**4.41(2.63-7.39)****
Urban	69(23.4%)	66(55.9)	1.00	1.00

**Occupation**				
Self-employed	219(74.2)	80(67.8)	1.37(0.86-2.18)	1.49(0.85-2.63)
Government employed	76(25.8)	38(32.2)	1.00	1.00
**Age**				
15-24	86(29.2)	29(24.6)	1.52(0.76-3.05)	1.26(0.56-2.80)
25-34	172(58.3)	70(59.3)	1.26(0.68-2.34)	1.34(0.66-2.72)
≥35	37(12.5	19(16.1%)	1.00	1.00
**Income**				
≤500	34(11.5)	7(5.9)	1.92(0.82-4.52)	1.69(0.64-4.42)
501-999	69(23.4)	35(29.7)	0.78(0.48-1.27)	0.94(0.53-1.68)
≥1000	192(65.1)	76(64.4)	1.00	1.00
**ANC follow up**				
No	146(49.5)	38(32.2)	2.06(1.32-3.23)	**2.62(1.54-4.48)*** **
Yes	149(50.5)	80(67.8)	1.00	1.00
**Birth attendant**				
Untrained attendant	161(54.6)	36(30.5)	2.74(1.74-4.31)	**2.67(1.58-4.51)******
Trained attendant	134(45.4)	82(69.5)	1.00	1.00
**Number of live birth**				
2-4	157(53.2)	70(59.3)	0.78(0.51-1.20)	0.69(0.41-1.14)
≥5	138(46.8)	48(40.7)	1.00	1.00
**Health facility accessibility**				
>5 km	150(50.8)	50(42.4)	1.41(0.92-2.16)	1.42(0.87-2.34)
≤ 5 km	145(49.2)	68(57.6)	1.00	1.00

*Significant with P<0.001, **Significant with P<0.001, ***Significant with P = 0.012 and ****Significant with P<0.002

Discussion

In this study, conducted to assess the level of cultural malpractices during perinatal period in southern Ethiopia, we found that 71.4% of participants were committed cultural malpractices. This finding suggests that health care providers should take into account the potential risk of

cultural malpractice while assessing clinical health assessment during antenatal care visit, childbirth ad post-natal visits of a woman. Besides, for healthcare planners this is vital. This knowledge can be used to build relevant programmes, channeling scarce resources to teaching what is needed as opposed to imparting messages that are already known.

This level of cultural malpractices is higher than what is reported from a study done elsewhere in Ethiopia and Cambodia [18, 20]. The discrepancy of these findings might be attributed to the difference in method used and study settings, sociodemographic characteristics of the study participants, and the availability and accessibility of the health services infrastructures. The report in this study implies that there is the lack of balance that the zone health office and regional health bureau could work in collaboration with the local health care giver to lessen the cultural malpractice of women during perinatal period.

This finding was lower than the study conducted in Northwest Ethiopia and Turkey [16, 21]. This difference can be explained by the difference in background of the study participants and method used and study settings, time gap, and the availability and accessibility of the infrastructures. In addition to this, the health system-related factors might contributing to this difference due to the extensive work of health extension workers and various health care institutions in awareness creation about the drawback of cultural malpractice in the study area. The finding implies that there is crated platform for maternity care providers that could help them to be aware of local values, beliefs and traditions to anticipate and meet the needs of women, gain their trust and work with them.

In this study we have found a number of factors associated with cultural malpractice during perinatal period. These included had no formal education, being a rural residence, had no ANC follow up for the last pregnancy and attended by untrained attendant.

Women who had no formal education were almost 4 times more likely performed cultural malpractice than women who had secondary education and above. Similar studies conducted in Nepal, Bangladesh and Northwest Ethiopia has found that education level is an important factor of cultural malpractice during perinatal period [7, 8, 18]. This may be related to as women did not attend formal education they could not easily understand the drawback of cultural malpractice on the health of the women themselves and their new born baby. Moreover, women who had no formal education will not have better awareness about the benefits of preventive

health care including avoiding cultural malpractice and lower receptivity to new health-related information.

Regarding, the association of residency with cultural malpractice during perinatal period in this study, those who have rural residency were nearly 5 times more likely executing cultural malpractice than those who have urban residency. This is in-line with the fact that women that are rural residence will not have information that could assist them in making decisions regarding healthy behaviors including maternal and child health education and promotion. Hence, women who have rural residency will have lack of access and availability of infrastructures like mass media and others that could enable them to be aware about the disbenefit of cultural malpractice during perinatal period. This is in line with a study conducted in Northern Ethiopia and North Karnataka [17, 23].

In this study, women who had no sought antenatal care during the last pregnancy were almost 3 times more likely carried out cultural malpractice than women who had sought antenatal care visit. The finding of this study was comparable with the findings of studies conducted in Ethiopia, UK and Taiwan [22, 24-25]. This could be due to the fact that women who had no previous ANC follow-up during the last pregnancy will not be aware on the drawback of cultural malpractice on the health of women and the fetus during perinatal period. In Western Region of Ghana, Traditional beliefs and practices as well as negative attitude of health workers are found to reduce health utilization by pregnant women. Hence, health education concerning traditional practices that are detrimental to the health of pregnant women should be emphasized during ANC visits [26].

Concerning, the association of attended by untrained attendant with cultural malpractice during perinatal period in this study, those who have attended by untrained attendant were almost 3 times more likely executing cultural malpractice than those who have attended by trained attendant. This is in-line with the fact that women that are attended by untrained attendant will not have contact with the healthcare provider in the health facility during perinatal period. Which in turn the women will not have information about the effect of cultural malpractice on health of the women themselves and their infants in the MCH clinic. The finding of this study is consistent with the studies done in Cambodia and elsewhere Ethiopia [20, 27-28].

Conclusion

Cultural malpractice was found to be high in the study area. In this study, had no formal education, being a rural residence, had no ANC follow up for the last pregnancy and attended by untrained attendant were factors significantly associated with cultural malpractice during perinatal period. Primary health care workers should take up the active role of women health education on the importance of ANC visit. Besides, apart from primary health care workers, other stakeholders in the maternal health sector should also create awareness among women on those services in which they could actively get MCH clinic. The government should come up with policies that are help to promote women formal education and child birth by trained attendant.

Declaration

List of abbreviation

ANC Antenatal Care

CMP Cultural Malpractice

MCH Maternal and child Health

NGO Non-Governmental Organization

SPSS Statistical package for social science

Funding

The authors have not declared a specific grant for this research from any funding agency in the public, commercial or not-for-profit sectors.

Patient consent for publication: Not required.

Availability of data and materials

The full data set and other materials about this study can be obtained from the corresponding author on reasonable request.

Competing interest

The author declares that there is no competing interest.

Authors' contribution

HA: Conceives the research idea, proposal development, supervised data collection process, conduct the analysis and wrote the manuscript.

GA: involved in proposal development, data analysis and wrote the manuscript.
BS: involved in data analysis, result writing and wrote the manuscript. All authors have read and approved the manuscript.

References

1. World Health Organization (WHO). Maternal Mortality Fact sheet. 2019; Available from: www.who.int/news-room/fact-sheets/detail/maternal-mortality.
2. Ethiopian Central Statistical Agency, C. and ICF, Ethiopia Demographic and Health Survey 2016: Key Indicators Report. 2016, CSA and ICF: Addis Ababa, Ethiopia, and Rockville, Maryland, USA.
3. United Nations (UN), Fact Sheet No.23, Harmful Traditional Practices Affecting the Health of Women and Children, UN Office of the High Commissioner for Human Rights (UNCHR), Editor. 2006: Geneva, Switzerland.
4. FDRE, M.o.W., Children and Youth Affairs (MoWCYA), National Strategy and Action Plan on Harmful Traditional Practices (HTPs) against Women and Children in Ethiopia, M.o.W. FDRE, Children and Youth Affairs (MoWCYA), Editor. 2013: Addis Ababa.
5. Adatara, P., et al., Cultural beliefs and practices of women influencing home births in rural Northern Ghana. International Journal of Women's Health 2019. **11** p. 353-361.
6. Tola, T.N. and A.H. Tadesse, Cultural Malpractices During Pregnancy, Child Birth and Postnatal Period Among Women of Child Bearing Age in Limmu Genet Town, Southwest Ethiopia. Science Journal of Public Health, 2015. **3**(5): p. 752-756.
7. Sharma, S., et al., Dirty and 40 days in the wilderness: Eliciting childbirth and postnatal cultural practices and beliefs in Nepal. BMC Pregnancy and Childbirth, 2016. **16**(147).
8. Choudhury, N., et al., Beliefs and practices during pregnancy and childbirth in urban slums of Dhaka, Bangladesh. BMC Public Health, 2012. **12**(791).
9. Hossain, K., et al., Beliefs and Practices During Antenatal and Postnatal Period Among the Host Communities in Cox’s Bazar, Bangladesh. American Journal of Biomedical Science & Research, 2020 **9**(6).
10. Sumankuuro, J., J. Crockett, and S. Wang, The use of antenatal care in two rural districts of Upper West Region, Ghana. PLoS One, 2017. **12**(9): p. e0185537.
11. Chakona, G. and C. Shackleton, Food Taboos and Cultural Beliefs Influence Food Choice and Dietary Preferences among Pregnant Women in the Eastern Cape, South Africa. Nutrients, 2019. **11**.
12. Zepro, N.B., Food Taboos and Misconceptions Among Pregnant Women of Shashemene District, Ethiopia. Science Journal of Public Health, 2015. **3**(3): p. 410-416.

13. Alene, G.D. and M. Edris, Knowledge, Attitudes and Practices involved in Harmful Health Behavior in Dembia District, northwest Ethiopia. Ethiop.J.Health. Dev, 2002. **16**(2): p. 199-207.
14. Gedamu, H., A. Tsegaw, and E. Debebe, The Prevalence of Traditional Malpractice during Pregnancy, Child Birth, and Postnatal Period among Women of Childbearing Age in Meshenti Town. International Journal of Reproductive Medicine, 2016.
15. Mesele, H.A., Traditional Maternal Health Beliefs and Practices in Southern Tigray: The Case of Raya Alamata District. Anatomy & Physiology: Current Research, 2018. **8**(2).
16. Zenebe, K., et al., Prevalence of cultural malpractice and associated factors among women attending mch clinic at debretabor governmental health institutions south Gondar, Amhara region, North West Ethiopia. Gynecology & Obstetrics, 2016. **6**(4).
17. Gebrekirstos, K., M. Abebe, and A. Fantahun, A cross sectional study on factors associated with harmful traditional practices among children less than 5 years in Axum town, north Ethiopia. Reproductive Health, 2014. **11**(46).
18. Gedamu, H., A. Tsegaw, and E. Debebe, The Prevalence of Traditional Malpractice during Pregnancy, Child Birth, and Postnatal Period among Women of Childbearing Age in Meshenti Town, 2016. International journal of reproductive medicine, 2018.
19. Federal Democratic Republic of Ethiopia Central Statistical Agency, C.E., Population Projection of Ethiopia for All Regions At Wereda Level from 2014 – 2017. 2013.
20 Claudia Turner, Sreymom Pol, Kamsan Suon, Leakhena Neou, Nicholas P. J. Day, Parker M, Kingori P: Beliefs and practices during pregnancy, post-partum and in the first days of an infant’s life in rural Cambodia. BMC Pregnancy and Childbirth 2017 17:116(10.1186/s):12884-12017-11305-12889.
21. Keno D: cultural practices during pregnancy & child birth among WCBA in shebe town. In.: research report summated to department of health officer as practical …; 1998.
22. Oni O, Tukur JJAJoRH: Nutritional taboos among pregnant women. 2012.
23. Nethra N, Udgiri R: A study on traditional beliefs and practices in newborn care among mothers in a tertiary health care centre in Vijayapura, North Karnataka. Int J Community Med Public Health 2018 :, 5((3)):1035-1040.

24. Fekede Asefa, Ayele Geleto, Dessie Y: Male partners involvement in maternal ANC care: The view of women attending ANC in Hararipublic health institutions, eastern Ethiopia. Science Journal of Public Health 2014, 2(3):182-188.
25. Huang Y-C: A comparative study of traditional postpartum practices and rituals in the UK and Taiwan. Diversity in Health and Care 2010, 7:239–247.
26. Patience Otoo, Helen Habib, Ankomah A: Food Prohibitions and Other Traditional Practices in Pregnancy: A Qualitative Study in Western Region of Ghana. Advances in Reproductive Sciences 2015, 3:41-49.
27. Getinet Kassahun, Negash Wakgari, Abrham R: Patterns and predictive factors of unhealthy practice among mothers during pregnancy, childbirth, postnatal and newborn care in Southern Ethiopia: a community based cross‑sectional study BMC Res Notes 2019, 12:594.
28. Sena Belina Kitila, Wondwosen Molla, Tilahun Wedaynewu, Tadele Yadessa, Melikamu Gellan: Folk Practice During Childbirth and Reasons for the Practice in Ethiopia: A Systematic Review. Gynecol Obstet , 2018, 8:3.
