# Supplementary material for: Harmful cultural practices during perinatal period and associated factors among women of childbearing age in Southern Ethiopia: Community based cross-sectional study
Source: PLoS One. 2021 Jul 2;16(7):e0254095. doi: 10.1371/journal.pone.0254095 (PMC8253409; doi:10.1371/journal.pone.0254095)
Supplement: Attachment — Submitted filename: Authors point by point responses.edited.docx [file pone.0254095.s004.docx]

**[Authors Response](https://journals.plos.org/plosone/article/peerReview?id=10.1371/journal.pone.0222843)**

Cultural malpractice during perinatal period and associated factors among women of child bearing age in Southern Ethiopia: Community based cross-sectional study

**Corresponding author: Haimanot Abebe/**[**haimanotabebe78@gmail.com**](mailto:haimanotabebe78@gmail.com)

**Authors**

^1^. Haimanot Abebe/[haimanotabebe78@gmail.com](mailto:haimanotabebe78@gmail.com)

^2^. Girma Alemayehu /[lgaglovely@gmail.com](mailto:lemmagetacher@gmail.com)

^3^. Berhanu Semere /brishsem@gmail.com

**Manuscript ID** PONE-D-20-34446

**Journal:** PLOS ONE

Article type: Research article

**Authors Point by point response to Reviewers and Editor**

First of all, the authors would like to thank “PLOS ONE” Journal editors and the respective reviewers for reviewing our manuscript and providing the necessary comments to be corrected and more scientifically acceptable. As per the comments given, we have made corrections in the revised version of the manuscript. The authors tried to answer all the issues which are presented as a question and raised by the editorial team and reviewers point by point. Accordingly, all comments are incorporated and accepted as comment in the revised document of manuscript.

**Point by point response to Editor**

Thank you very much, Dear **Sir/Madam (Editor of** PLOS ONE **Journal)**, for your in-depth, valuable and concrete comments that you provided. Generally, we would like to praise you for your help and work to make our manuscript highly scientifically sound. Accordingly, we have corrected all the comments you provided in the revised manuscript.

**Editors: -** Please ensure that your manuscript meets PLOS ONE's style requirements, including those for file naming. The PLOS ONE style templates can be found at.

**Authors Response:** **Dear Nülüfer Erbil** **[Ph.D, Prof.Academic (Editor PLOS ONE)]**, Thank you very much for your constructive comments. We have taken correction in the revised manuscript accordingly.

**Editors: -** Please include additional information regarding the survey or questionnaire used in the study and ensure that you have provided sufficient details that others could replicate the analyses. For instance, if you developed a questionnaire as part of this study and it is not under a copyright more restrictive than CC-BY, please include a copy, in both the original language and English, as Supporting Information.

**Authors Response:** **Dear Nülüfer Erbil** **[Ph.D, Prof.Academic (Editor of PLOS ONE)]**, thank you very much; we incorporate information regarding the survey or questionnaire in both the original language and English in the revised manuscript.

**Editors:** Thank you for stating the following financial disclosure: "The funders had no role in study design, data collection and analysis, decision to publish, or preparation of the manuscript." At this time, please address the following queries:

a. Please clarify the sources of funding (financial or material support) for your study. List the grants or organizations that supported your study, including funding received from your institution.
b. State what role the funders took in the study. If the funders had no role in your study, please state: “The funders had no role in study design, data collection and analysis, decision to publish, or preparation of the manuscript.”
c. If any authors received a salary from any of your funders, please state which authors and which funders.
d. If you did not receive any funding for this study, please state: “The authors received no specific funding for this work.”
e. Please include your amended statements within your cover letter; we will change the online submission form on your behalf.

**Authors Response:** **Dear Nülüfer Erbil** **[Ph.D, Prof.Academic (Editor of PLOS ONE)]**, thank you very much; we have corrected the financial disclosure accordingly in the revised manuscript.

**Editors:** We note that you use the term "cultural malpractice" throughout your manuscript and title. As the term "malpractice" is tied to specific legal meanings, we request that you change this term throughout the manuscript to something that does not contain the word "malpractice".

**Authors Response:** **Dear Nülüfer Erbil** **[Ph.D, Prof.Academic (Editor of PLOS ONE)]**, thank you very much; we have made modification accordingly in the revised manuscript.

**Point by point response to (Reviewer # 1)**

**Thank you very much, Dear Sir/Madam (Reviewer # 1), we would like to praise you for your help and work to make our manuscript is highly scientifically sound. Saying this, we have corrected all the comments you provided in the revised manuscript. Hence, comments are accepted and incorporated in the revised manuscript. On the other hand, comments are not written in the point by point response. Therefore, we are only given answers for the question you raised one by one.**

**Reviewer # 1:** The necessary corrections for the article are shown on the text. Please review the abstract section. There is an inconsistency in your sample size. I can only write in one language and admire anyone who attempts to write in a language other than their native one. Unfortunately the English used here sometimes obscures your meaning. There is incorrect use of the definite and indefinite article, tenses, spelling and plural/singular to name a few problems. The manuscript should have been reviewed by a native English speak.

**Authors Response:** Thank you very much**, Dear Sir/Madam (Reviewer # 1),** we have taken correction for the comments given accordingly in the revised manuscript. And we are also reviewed and solved all iniquities related with the English language.......including the tense used and unnecessary capitalization and other typos/ errors by a person who had PHD in English.

**Reviewer # 1:** You wrote 422 in the sample size part, 413 women in the findings. What happened to 9 women?

**Authors Response:** Thank you very much, Dear Sir/Madam (Reviewer # 1), the discrepancy for 422 sample size and 413 women is emanated from the non-response rate. Hence, 9 individuals were non-response rate; this was made when the respondents are not available at home at the first visit, a revisit was arranged at a minimum of three times. Then we call them non-respondents if they were not found after three hose visit. That is why the difference is happened.

**Point by point response to Pauline Williams (Reviewer # 2)**

**Thank you very much, Dear Alex Yeshaneh, for your in-depth, valuable and concrete comments that you provided. In nutshell, we would like to praise you for your help and work to make our manuscript is highly scientifically acceptable. Saying this, we have corrected and accepted all the comments you provided in the revised manuscript.**

**Reviewer # 2:** Keywords should be no more than 5 and no less than 3.
“Perinatal period is considered as time period including the pregnancy, childbirth and postnatal period” what is implication of stating term definition in abstract?

**Authors Response:** Thank you very much, **Dear Alex Yeshaneh,** we have made correction accordingly in the revised manuscript.

**Reviewer # 2**:- Your method in abstract lacks overview of sampling technique, data presentation and analysis.

**Authors Response:** Thank you very much, **Dear Alex Yeshaneh,** we have made correction accordingly in the revised manuscript and we have incorporated.

**Reviewer # 2**:- As far as this study has two objectives, it should have to consider sample size calculation for second objective too.

Authors Response: Thank you very much, **Dear Alex Yeshaneh**, Yes of course we have to calculate the simple size for the two objectives as far as it is two objectives. But, when we determine the sample size for the two objectives we were found the maximum sample size in the first objective. That is why we used the sample size calculated for the 1^st^ objective.

**Reviewer # 2**:- “Perinatal period: Time period including the pregnancy, childbirth and postnatal period” is term definition or operational definition? Clarify?

**Authors Response:** Thank you very much, **Dear Alex Yeshaneh,** we have made correction accordingly in the revised manuscript. “Perinatal period: as it was a term definition, we have taken correction.

**Reviewer # 2**:- “The data collected were entered into Epi Info version ™ 7 and exported to Statistical Package for Social Science (SPSS) version 25 for cleaning and analysis” why? Cleaning and analysis was also possible for Epi info.

**Authors Response:** Thank you very much, **Dear Alex Yeshaneh,** of course we can use both for data entry and analysis, but “EpiInfo” are good for data entry • because it is so fast, reliable, allows for controlled data-entry, on the other hand SPSS is good statistical software for data analysis. That is why in most case, investigators prepare their data with data management software and transfer to statistical software for analysis. So then, we used EpiInfo for data management to be benefited for data control, and SPSS for statistical analysis.

**Reviewer # 2**:- You stated multivariate and multivariable analysis interchangeably. Do you think it is similar? Which one you used? Justify?

**Authors Response:** Thank you very much, **Dear Alex Yeshaneh,** we have made correction accordingly in the revised manuscript. We used multivariable which can examine three or more variables at a time. Those analyses has their own difference, hence multivariate analysis enables you to avoid the problem of multiple tests that would arise if you tested the effect of each independent variable on each dependent variable separately whereas multivariable analysis enable you when there is more than one predictor variable in a multivariate regression model.

**Reviewer # 2** “The mean age of study participants were 27.6 (SD ± 5.4 years)”. Did you check normality test? If yes how? Why you select mean to describe age? Why didn’t other descriptive statistics?

**Authors Response:** Thank you very much, **Dear Alex Yeshaneh,** yes we have made normality for selected variables especially for continuous variable by using Graphic method-Histogram line plot, numeric method –descriptive: skewness, kurtosis. And we use the mean which is the most popular measure and very easy to calculate. It is a unique value for one group, that is, there is only one answer, which is useful when comparing between the groups. In the computation of mean, all the observations are used.

**Reviewer # 2**:- Are these ethnic groups only dwell in the study area?

**Authors Response:** Thank you very much, **Dear Alex Yeshaneh,** no the study setting is a palace where many ethnic group have found. But, unfortunately we have got the response of three ethnic group.

**Reviewer # 2**:- “Koso”, “telba”… is local language and it should have to be elaborated and italic while you write.

**Authors Response:** Thank you very much, **Dear Alex Yeshaneh,** we have taken correction accordingly in the revised manuscript.

**Reviewer # 2**:- In table 2, Frequency of food taboo is beyond total sample size? Do you have a justification? Why pre-lacteal feeding percentage left blank? Justify?

**Authors Response:** Thank you very much, **Dear Alex Yeshaneh,** we have taken correction accordingly in the revised manuscript. And it was typing errors.

**Reviewer # 2**:- “Regarding ANC follow up, participants who had ANC follow up during the last pregnancy were almost three [AOR 2.62, 95%CI, 1.54-4.48] times more likely execute cultural malpractice during perinatal period.” How do you justify this? It contradicts the logic and the science? Is there possible explanation for your study?

**Authors Response:** Thank you very much, **Dear Alex Yeshaneh,** we have taken correction accordingly in the revised manuscript. This was typing error and it was to say the vice versa of the written one.

**Reviewer # 2**:- Food taboo is a vague word. How you assessed it?

**Authors Response:** Thank you very much, **Dear Alex Yeshaneh,** food taboo is abstaining women from food and/or beverage consuming due to religious and cultural reasons during perinatal period.

**Reviewer # 2**:- “This level of cultural malpractices is higher than what is reported from a study done elsewhere in Ethiopia” and “This finding was lower than the study conducted in Northwest Ethiopia” these two sentences are contradicting? Justify?

**Authors Response:** Thank you very much, **Dear Alex Yeshaneh,** we have taken correction accordingly in the revised manuscript to make the reader clearer.

**Reviewer # 2**:- Your study was community based cross-sectional study and asks retrospectively about perinatal period. This is prone your study to recall bias. How did you reduce recall bias for this study?

**Authors Response:** Thank you very much, **Dear Alex Yeshaneh,** the study might be subjected to recall bias because the mothers failed to remember what they did during perinatal period. This was minimized by probing the respondents about the event.

**Reviewer # 2**:- Is your study had no limitation? If yes add limitations of the study.

**Authors Response:** Thank you very much, **Dear Alex Yeshaneh,** We add the limitation of the study in the revised manuscript.

**Reviewer # 2**:- Reference no.14 and no.18 are similar. Consider revision

**Authors Response:** Thank you very much, **Dear Alex Yeshaneh,** we have taken correction accordingly in the revised manuscript.

**Point by point response to Pauline Williams (Reviewer # 3)**

**Thank you very much, Shireen Shehzad Bhamani, for your in-depth, valuable and concrete comments that you provided. In nutshell, we would like to praise you for your help and work to make our manuscript is highly scientifically acceptable. Saying this, we have corrected and accepted all the comments you provided in the revised manuscript. As well as we provided a point by point response for your questions.**

**Reviewer # 3:-** It was good to see researchers are trying to address an important issue of developing country

**Authors Response:** Thank you very much, **Shireen Shehzad Bhamani,** we have enlisted significant issues related to cultural malpractice during perinatal period specifically in the developing world in the revised manuscript.

**Reviewer # 3**:- It would be appreciated, if author can explain the reasons of repeating the similar study in different zone of Ethiopia, when it was already conducted in North West Ethiopia (mentioned in sample size reference).

### Authors Response: - Thank you very much, Shireen Shehzad Bhamani, the reason for doing this study in different zone of Ethiopia specifically in Gurage zone, despite having a few studies done in Ethiopia, there is paucity of data concerning cultural malpractice during perinatal period and associated factors among women of child bearing age in Ethiopia particularly there is a dearth of study in Southern region of Ethiopia specifically in Gurage Zone. There are also inconstancies of findings between studies done in different zone of Ethiopia on prevalence and factors associated with cultural malpractice. Besides, there was a methodological limitations which have been conducted before in Ethiopia( for instance, there was a different in study population, small sample size, done in specific places not in Zonal level, and there was a difference in study design which is mainly focused in institutional based cross-sectional studies). List of studies reference with sample size include in Meshenti town, Amhara region, northwest Ethiopia: There were 316 study subjects, in Debretabor Governmental Health Institutions South Gondar, Amhara Region, North West Ethiopia. There were 355 study subjects, in Limmu Genet Town, Oromia Region, There were 303 study subjects. And mainly we are interested as well because of the region is containing different ethnicity groups which make the region different from other region of Ethiopia. Here, for example in this zone (In Gurage zone Amhara, Oromo and Wolayita are found). Therefore, we are come up with this study by overcome those of the limitations which are listed above.

**Reviewer # 3:-** It would be nice, if STROBE guidelines can be followed so that methodology can be fully evaluated.

**Authors Response:** Thank you very much, **Shireen Shehzad Bhamani,** we included in the revised manuscript.

**Reviewer # 3:-** The whole document needs a substantial language revision to improve grammar and style.

**Authors Response:** Thank you very much, **Shireen Shehzad Bhamani,** we have made correction in the revised manuscript.

**Reviewer # 3:-** The whole document should be revised considering the within text citation as it has been noticed at multiple places where references within text references are missing like page 3, paragraph 1, line 2 and 6 and paragraph 5 line 2, 4 etc.

**Authors Response:** Thank you very much, **Shireen Shehzad Bhamani,** we have made correction in the revised manuscript.

**Reviewer # 3:-** Study setting needs further description in terms of population being catered like their ethnicity, socio economic status so one can understand the homogeneity or heterogeneity among the settings

**Authors Response:** Thank you very much, **Shireen Shehzad Bhamani,** we have made correction in the revised manuscript.

**Reviewer # 3:-** Kindly justify why are you calling the sampling technique as simple random sampling and not proportionate or multi-level sampling

**Authors Response:** Thank you very much, **Shireen Shehzad Bhamani,** since the study area found in the zone are homogenous in affecting the outcome variable and a simple random sample is used to represent the entire data population as well as simple random sampling does not require additional information on the frame (such as geographic areas) and the formulas are easy to use we select simple random sampling technique. On the other hand portionate (Stratified random sampling) is a method of probability sampling in which the population is divided into different subgroups and samples are selected from each subgroup and used mainly for , the population is considered to be heterogeneous (having significant variation among individuals). Unlike simple random samples, stratified random samples are used with populations that can be easily broken into different subgroups or subsets.  Multi-stage sampling method is appropriate when the reference population is large and widely scattered. Selection is done in stages until the final sampling unit (eg. households or persons) are arrived at.

**Reviewer # 3:-** How did you maintain the consistency between different data collectors? As there were 6 separate data collectors

**Authors Response:** Thank you very much, **Shireen Shehzad Bhamani,** we have provided training for data collectors regarding the objective of the study, data collection tool, ways of data collection, checking the completeness of data collection tool and how to maintain confidentiality. So then they were acquiring a good knowledge about data collection. Moreover, the principal investigator, supervisors and the data collectors were a meet together for a discussion about the data collection process every day after data collection so that during this time we were discussed about the activity and the challenges and problems faced during their time of data collection every day, and we solved together what they faced. As a result we have maintained the consistency between different data collectors during the data collection period.

**Reviewer # 3:-** Who did training for data collectors and what was covered in training?

**Authors Response:** Thank you very much, **Shireen Shehzad Bhamani,** all the investigators are provided enough training for all data collectors, and the training covered the objective of the study, data collection tool, ways of data collection, checking the completeness of data collection tool and how to maintain confidentiality.

**Reviewer # 3:-** Authors have mentioned that corrections and modification was made after pretesting, please specify

**Authors Response:** **confounding variable:**  Thank you very much, **Shireen Shehzad Bhamani,** the questionnaire was pretested a week before the actual survey in comparable setting in Silte town on 5% of the calculated sample size, after which the necessary correction and modification was made accordingly. Fore stance, participants were asked about the travel time to health institution but they were not familiarized about this. Instead, they were express the distance between their home and health institution. Accordingly, we have changed the question that says “What is the time taken to reach to health institution” into “what is the distance between your home and the institution in Kilometer”. And we also tried to short and brief a too long question.

**Reviewer # 3:-** Why the results considered confounding variable? As this study does not have any main exposure so please share the reasons of considering confounders as it is not meeting the criteria of assessing confounders.

Authors Response: Thank you very much, Shireen Shehzad Bhamani, as we know in research that investigates a potential cause-and-effect relationship, is an unmeasured third [variable](https://www.scribbr.com/methodology/types-of-variables/) that influences both the supposed cause and the supposed effect. It’s important to consider potential confounding variables and account for them in your [research design](https://www.scribbr.com/research-process/research-design/) to ensure your results are [valid](https://www.scribbr.com/methodology/internal-vs-external-validity/). Thus, if you see the association between the independent variable and the outcome variable of this study such as Educational status and cultural malpractice, education is one variable that affects the cultural malpractice. On the other hand residence is one factor that determines cultural malpractice; similarly, residence might affect the educational status of the respondent. Therefore, to overcome this potential confounder we mad multivariable analysis. Because confounder variable could cause two major problems including **introducing bias and increasing variances we made multivariable analysis.**

**Reviewer # 3:-** did the author assess the normality of each variable?

**Authors Response:** **confounding variable:**  Thank you very much, **Shireen Shehzad Bhamani,** yes we have made normality tests for variables by using graphically and numerically methods. Therefore, to test the normal distribution of the data we used those of the following:-

### Normal Q-Q Plot: In order to determine normality graphically, we can use the output of a normal Q-Q Plot. If the data are normally distributed, the data points will be close to the diagonal line. If the data points stray from the line in an obvious non-linear fashion, the data are not normally distributed. As we can see from the normal Q-Q plot below, the data is normally distributed. If you are at all unsure of being able to correctly interpret the graph, rely on the numerical methods instead because it can take a fair bit of experience to correctly judge the normality of data based on plots.

**Histogram**: When a histogram’s shape approximates a bell-curve it suggests that the data may have come for a normal population.

**Kolmogorov–Smirnov test**: (**K–S test** or **KS test**) is a [nonparametric test](https://en.wikipedia.org/wiki/Nonparametric_statistics) of the equality of continuous. quantifies a [distance](https://en.wikipedia.org/wiki/Metric_(mathematics)) between the [empirical distribution function](https://en.wikipedia.org/wiki/Empirical_distribution_function) of the sample and the [cumulative distribution function](https://en.wikipedia.org/wiki/Cumulative_distribution_function) of the reference distribution, or between the empirical distribution functions of two samples. If the **Sig.** value of the Shapiro-Wilk Test is greater than 0.05, the data is normal. If it is below 0.05, the data significantly deviate from a normal distribution.

**Shapiro**-**Wilk Test:** value of the Shapiro-Wilk Test is greater than 0.05, the data is normal. If it is below 0.05, the data significantly deviate from a normal distribution. If you need to use skewness and kurtosis values to determine normality, rather the Shapiro-Wilk test, you will find these in our enhanced testing for normality guide.

**Reviewer # 3:-** data results were based on 413 whereas in abstract it was mentioned 422. Please correct this and explain the reasons of dropout?

**Authors Response:** Thank you very much, **Shireen Shehzad Bhamani,** the discrepancy for 422 sample size and 413 women is emanated from the non-response rate. Hence, 9 individuals were non-response rate; this was made when the respondents are not available at home at the first visit, a revisit was arranged at a minimum of three times. Then we call them non-respondents if they were not found after three hose visit. That is why the difference is happened.

**Reviewer # 3:-** did researcher did the stratified analysis on different perinatal time i.e pregnancy, childbirth & post-natal? This would be a great addition to see at which phase culture malpractices affects the most.

**Authors Response:** Thank you very much, **Shireen Shehzad Bhamani,** of course it is good but we are focused to assess the overall magnitude of cultural malpractice as a whole not in single way that would imply for police makers and other concerned body which probably help to improve the health of the women and child. As well since previous studies conducted in Ethiopia are investigated in a stratified way we are to plan to study in similar circumstances.

**Reviewer # 3:-** On Page 9 in table 2, percentages are missing for pre lacteal feeding.

**Authors Response:** Thank you very much, **Shireen Shehzad Bhamani,** we have taken correction accordingly in the revised manuscript.

**Reviewer # 3:-** Does researcher assess multi collinearity between independent variables?

**Authors Response:** Thank you very much, **Shireen Shehzad Bhamani,** yes we have fix the problem of multicollinearity that may occurs when there is a high correlation between the independent variables in the regression analysis which impacts the overall interpretation of the results. It reduces the power of coefficients and weakens the statistical measure to trust the p-values to identify the significant independent variables. Hence, we would not be able to examine the individual explanation of the independent variables on the dependent variable. Therefore, in this study the multicollinearity problem was detected with the help of tolerance and its reciprocal, called variance inflation factor (VIF). Hence, variable with a value of tolerance is less than 0.1 and, simultaneously, the values of VIF 10 and above were checked accordingly. Unfortunately, no variable was detected that has collinearity effect or variable which has tolerance test less than <0.1 and VIF>10.

**Reviewer # 3:-** Can these finding be generalized, justify and write yes or no with proper justification.

**Authors Response:** Thank you very much, **Shireen Shehzad Bhamani,** yes this finding is a generalized for the whole population in the study setting because in this study we were taken 30-40% of the total woreda in the study setting based on WHO representatives or generalizability. Hence, this study meets the criteria of world health organization criteria of representatives. In addition to this, as far as we used a simple random sampling technique and the aim of a study is to measure variables distributed in a population or to test hypotheses about which factors are contributing significantly to a certain problem, we have to be sure that we can generalize the findings obtained from a sample to the total study population.

**Reviewer # 3:-** Author should highlight the strengths and limitation of this study? Any bias ( selection or reporting) observed & how it was managed?

**Authors Response:** Thank you very much, **Shireen Shehzad Bhamani,** we included the strength and limitation of the study in the revised manuscript. And we were managed a bias like recall bias through probing the respondents about the event which happened a month’s back. Here, we were performed activities to overcome a bias like a selection bias which comes from any error in selecting the study participants and/or from factors affecting the study participation. Good researchers will look for ways to overcome selection bias in their observational studies. Therefore, we were tried to make our study representative by including as many people as possible and by using a simple random sampling technique. Similarly, reporting bias which is defined as "selective revealing or suppression of information" by subjects, and managed through understanding of the importance of the information by the researchers.

***Thanks Again!!!***
