# Supplementary material for: Harmful cultural practices during perinatal period and associated factors among women of childbearing age in Southern Ethiopia: Community based cross-sectional study
Source: PLoS One. 2021 Jul 2;16(7):e0254095. doi: 10.1371/journal.pone.0254095 (PMC8253409; doi:10.1371/journal.pone.0254095)
Supplement: Attachment — Submitted filename: Authors point by point responses.edited.docx [file pone.0254095.s005.docx]

**[Authors Response](https://journals.plos.org/plosone/article/peerReview?id=10.1371/journal.pone.0222843)**

**Cultural harmful practices during perinatal period and associated factors among women of childbearing age in Southern Ethiopia: Community based cross-sectional study.**

**Corresponding author: Haimanot Abebe/**[**haimanotabebe78@gmail.com**](mailto:haimanotabebe78@gmail.com)

**Authors**

^1^. Haimanot Abebe/[haimanotabebe78@gmail.com](mailto:haimanotabebe78@gmail.com)

^2^. Girma Alemayehu Beyene /[lgaglovely@gmail.com](mailto:lemmagetacher@gmail.com)

^3^. Berhanu Semere Mulat /brishsem@gmail.com

**Manuscript ID** PONE-D-20-34446

**Journal:** PLOS ONE

Article type: Research article

**Author’s point by point response to Editor and Reviewers**

First of all, the authors would like to provide extremely gratitude to “PLOS ONE” Journal editors and the respective reviewers for reviewing our manuscript and providing important and pertinent comments to be corrected and more scientifically respectable and fairish. As per the comments given, we have made corrections in the revised version of the manuscript. Accordingly, all comments are incorporated and accepted as comment in the revised document of manuscript.

**Point by point response to Editor**

**Thank you very much, Dear Sir/Madam (Editor of PLOS ONE Journal),** for your all-inclusive, comprehensive, valuable and concrete comments that you provided. Generally, we would like to praise you for your unlimited help and work to make our manuscript highly scientifically acceptable. Accordingly, we have corrected all the comments you provided in the revised manuscript.

**Editors: -** Please review your reference list to ensure that it is complete and correct. If you have cited papers that have been retracted, please include the rationale for doing so in the manuscript text, or remove these references and replace them with relevant current references. Any changes to the reference list should be mentioned in the rebuttal letter that accompanies your revised manuscript. If you need to cite a retracted article, indicate the article’s retracted status in the References list and also include a citation and full reference for the retraction notice.

**Authors Response:** **Dear Nullifier Erbil [(PHD, Prof.) Academic Editor of PLOS ONE]**, Thank you very much for your formative and productive comments. We have taken correction in the revised manuscript accordingly. Outdated references are omitted from the references lists and replaced by recently published articles or literatures. Accordingly the following outdated references are changed.

1, United Nations (UN), Fact Sheet No.23, Harmful Traditional Practices Affecting the Health of Women and Children, UN Office of the High Commissioner for Human Rights (UNCHR), Editor. 2006: Geneva, Switzerland. **Is changed by** Foad HS, Katz R, Migration IO for. World Migration Report 2020 (full report) [Internet]. Vol. 54, European Journal of Political Research Political Data Yearbook. 2015. 1–18 p.

2. Alene, G.D. and M. Edris, Knowledge, Attitudes and Practices involved in Harmful Health Behavior in Demba District, northwest Ethiopia. Ethiop.J.Health. Dev, 2002. **16**(2): p. 199-207. **Is changed by** Hadush Z, Birhanu Z, Chaka M, Gebreyesus H. Foods tabooed for pregnant women in Abala district of Afar region, Ethiopia: An inductive qualitative study. BMC Nutr. 2017;3(1):1–9.

3, Keno D: cultural practices during pregnancy & childbirth among WCBA in shebe town. In.: research report summated to the department of health officer as practical …; 1998. **Is changed by** Nana A, Zema T. Dietary practices and associated factors during pregnancy in northwestern Ethiopia. BMC Pregnancy Childbirth. 2018;18(1):1–8.

**XXXX-** Concerning English language and grammatical editing suggested by the reviewers. We have taken corrections concerning to any English errors and typos by the help of a man who have PHD in English language. Furthermore, we have also taken correction for any English errors using online grammar and English typo correctors apps (We used the following links-

[https://app.grammarly.com/?network=g&utm_source=google&matchtype=e&gclid=Cj0KCQjwo-aCBhC-ARIsAAkNQiuJ49UHhl6ibhQfzq9D4wGrbSOeZPv49UoRqSnd4ThQ-KKrPp uBp4aAjLgEALw_wcB&placement=&q=brand&utm_content=486649398671&gclsrc=aw.ds&utm_campaign=brand_f1&utm_medium=cpc&utm_term=grammarly](https://app.grammarly.com/?network=g&utm_source=google&matchtype=e&gclid=Cj0KCQjwo-aCBhC-ARIsAAkNQiuJ49UHhl6ibhQfzq9D4wGrbSOeZPv49UoRqSnd4ThQ-KKrPp%20uBp4aAjLgEALw_wcB&placement=&q=brand&utm_content=486649398671&gclsrc=aw.ds&utm_campaign=brand_f1&utm_medium=cpc&utm_term=grammarly)

and

<https://pubsure.researcher.life/author/?active_tab=recent_plan>

So, now we have solved all iniquities related with the English language.......including the tense used and unnecessary capitalization and other typos/ errors

**Point by point response to (Reviewer # 3)**

**Thank you very much, Dear Sir/Madam (Reviewer # 3),** we would like to give you our appreciation or thankfulness for your endless support to make our manuscript scientifically well-conditioned. Besides, we would like to thank you for sharing your knowledge and experiences in the whole reviewing of this manuscript.

**Reviewer # 3:** I strongly feel that, study flow chart will be a great addition to this manuscript and it will clarify the sample enrolled or drop out at each phase.

**Authors Response:** **Thank you very much, Dear Sir/Madam (Reviewer # 1)**, we have taken correction accordingly in the revised manuscript.

**Reviewer # 3:** Some of the comments were appropriately answered by author but I could not see these were incorporated in manuscript like normality assessment, training details and steps taken by researchers to improve consistency between data collectors. Plus considering multicolinearity ( do share which variables were found correlated) etc.

**Authors Response:** **Thank you very much, Dear Sir/Madam (Reviewer # 3),** we have taken correction and incorporated all suggestion given accordingly in the revised manuscript.

***Thanks Again!!!***
